# IFRS 9 and procyclicality of loan loss provision among Chinese regional banks, the role of local leaders’ turnover

**DOI:** 10.1371/journal.pone.0336156

**Published:** 2025-11-19

**Authors:** Jiannan Yu, Maizatulakma Abdullah, Hamezah Md Nor

**Affiliations:** 1 School of Finance and Economics, Guangdong University of Science and Technology, Dongguan, Guangdong, China; 2 Faculty of Economics and Management, Universiti Kebangsaan Malaysia, Bangi, Selangor, Malaysia; 3 Faculty of Economics and Management, Universiti Kebangsaan Malaysia, Bangi, Selangor, Malaysia; BeiHang University School of Economics and Management, CHINA

## Abstract

This research aims to investigate the impact of IFRS 9 adoption on the procyclicality and the role of the local leaders’ turnover in this relationship. The financial accelerator theory and institutional theory provide a theoretical basis for this research. Using the panel data of 175 Chinese regional commercial banks from 2019–2022, this research estimates fixed-effects regression models to compare the procyclicality under IAS 39 and IFRS 9. The results reveal that the adoption of IFRS 9 mitigated procyclicality. This provides additional empirical evidence to the mixed results of prior studies, which were based on European countries. Further, the result also indicates that the local leaders’ turnover hinders the countercyclical effect of IFRS 9. This suggests that despite IFRS 9 helping alleviate procyclicality, the presence of local leaders’ turnover impedes achieving the countercyclical objective. These results highlight the importance of stable local leadership to the countercyclical function of IFRS 9. This research extends the geographical scope of research on IFRS 9. It is the first research that investigates the relationship between IFRS 9 adoption and the procyclicality in a non-Euro country. This research also provides insights into the interplay between IFRS 9, procyclicality, and local leaders’ turnover, and reveals the effect of political institutions on accounting practice. Additionally, this research contributes to the financial accelerator theory and institutional theory by extending their application into the accounting field. Based on these findings, this research recommends measures to enhance policy continuity during political transitions, strengthen forward-looking data infrastructure, improve supervisory oversight of discretionary provisioning, and tailor prudential policies.

## Introduction

During the global financial crisis in 2008, the International Accounting Standard 39 – Financial Instruments: Recognition and Measurement (IAS 39) suffered a lot of criticism. Most scholars and practitioners believed the loan loss provisions (LLP) under IAS 39 have a high degree of procyclicality and fueled the financial crisis [1–5]. During the economic booms, low LLP contributes to higher earnings and capital adequacy, which stimulates banks to improve their risk-taking and loan granting [6,7]. The increased loans facilitate firms’ investment and business extension, further fueling the economic growth [7]. Conversely, during the economic downturns, banks’ LLP increase due to the deteriorating credit quality of their loan portfolio, putting the banks’ capital under pressure, force banks to reduce the loan granting, and deepening recessions [8]. By synchronizing LLP with economic fluctuations, IAS 39 embedded this destabilizing feedback loop into bank behavior [9–12].

To address these deficiencies, the International Accounting Standards Board introduced IFRS 9 in 2014, replacing the backward-looking ICL model with the forward-looking expected credit loss (ECL) model [13–15]. By recognizing provisions before losses occur, IFRS 9 sought to break the tight linkage between LLP and the business cycle, reducing procyclicality and strengthening financial stability. However, while prior research has examined IFRS 9’s effects on provisioning, reporting, and lending [1,16–23], relatively little is known about its influence on procyclicality, especially in institutional environments where political factors shape local economic conditions, which are key inputs to ECL estimates.

This research addresses this gap by examining whether IFRS 9 reduces the procyclicality of LLP in Chinese local commercial banks, while explicitly considering the moderating role of local government leadership turnover. China’s province-focused banking structure enables a direct link between bank LLP and local economic cycles, while frequent leadership changes offer a unique opportunity to explore how political dynamics interact with accounting standards.

Using panel data from 175 Chinese regional commercial banks spanning 2019–2022, this research employs fixed-effects regression models to examine differences in procyclicality under IAS 39 and IFRS 9. The findings show that adopting IFRS 9 effectively mitigates procyclicality, offering new empirical evidence that complements the mixed results of previous studies conducted in European contexts. Moreover, the analysis reveals that turnover among local leaders weakens the countercyclical effect of IFRS 9, suggesting that while IFRS 9 can reduce procyclicality, frequent leadership changes hinder its full countercyclical potential. These results underscore the critical role of stable local leadership in realizing the countercyclical objectives of IFRS 9.

The contributions of this research are fourfold. First, this research extends IFRS 9 literature from bank-level outcomes to macroeconomic implications. Second, it demonstrates how institutional settings, particularly political leadership, can influence accounting standard effectiveness. Third, it offers policy insights for other emerging economies facing similar governance and structural challenges. Finally, by integrating Financial Accelerator Theory and Institutional Theory, it provides a theoretical basis for understanding how accounting standards and political factors jointly shape the credit cycle and, by extension, economic stability.

The reminder of the research are as follows, the second section is the institutional background of China, the third section is the literature review, the fourth section is theoretical framework, the fifth section develops the hypothesis, the sixth section is the research design, the seventh section is the empirical results, the eighth section is the robustness check, and the nineth section is the conclusion of the research.

### Institutional backgrounds of China

#### IFRS convergence and IFRS 9 adoption.

China has pursued a strategy of convergence with IFRS since 2006 [24]. Following the launch of IFRS 9, the China Accounting Standards (CAS) 22 – Financial Instruments: Recognition and Measurement, CAS 23 – Transfer of Financial Assets, and CAS 24 – Hedge Accounting were developed in alignment with IFRS 9. Because their content was identical to IFRS 9, they became known as the “Chinese version of IFRS 9” [25].

IFRS 9 comprises three key components. First, it introduces a new approach to the recognition, classification, and measurement of financial instruments. Second, it incorporates the expected credit loss (ECL) model. Third, it addresses hedge accounting [26]. Among these, the shift from the incurred loss model to the ECL model is regarded as a pivotal development [27]. Unlike the incurred loss recognition method, the ECL model takes a forward-looking approach, allowing banks to recognize expected credit losses either over the next 12 months or across the lifetime of a financial instrument [14]. Estimations must be based on past events, current conditions, and future economic expectations. This forward-looking design ensures that loan loss provisions are recognized as early as possible, thereby enhancing their ability to reflect changes in credit risk. Consequently, the ECL model is expected to reduce the impact of banks’ procyclical provisioning practices on the economic cycle [13].

More specifically, the ECL model operates through a three-stage mechanism that classifies financial instruments according to credit risk [28]. At initial recognition, instruments are placed in Stage 1, requiring provisions to cover the next 12 months. When the credit risk of a Stage 1 instrument increases significantly, it moves to Stage 2, where provisions must cover the lifetime expected credit loss. At this stage, interest is calculated on the total amount of the asset, meaning provisions are not deducted from the asset’s value. If a loss event occurs that resembles default, the instrument migrates to Stage 3. Provisions at this stage also cover lifetime expected credit losses; however, unlike Stage 2, interest is calculated based on the net amount of the financial instrument. Stage 3 instruments are conceptually similar to the incurred loss approach under IAS 39 [29].

The three-stage model improves the timeliness of loan loss provisions (LLPs). Under IAS 39, financial instruments were not classified into stages, and provisions were recognized only after objective evidence of impairment had occurred [30]. This often meant that low-risk assets suddenly became defaulted once such evidence appeared, resembling a direct jump from a Stage 1–like status to a Stage 3–like status under IFRS 9. As a result, provisions would increase sharply during economic downturns, moving in lockstep with the cycle and exhibiting strong procyclicality. In contrast, IFRS 9’s three-stage model requires management to recognize provisions earlier by introducing Stage 2 between Stages 1 and 3. When the future credit risk of Stage 1 assets increases significantly, they are reclassified into Stage 2, and lifetime expected credit losses must be recognized. This mechanism facilitates timely recognition of emerging credit issues and prevents delayed recognition of losses. During downturns, LLPs can therefore be drawn in advance, reducing the synchronicity between provisions and the economic cycle and mitigating procyclicality [29].

The transition from IAS 39 to IFRS 9 represents a major shift in accounting standards, with profound implications for accounting practices—particularly in banks, given their extensive financial instrument holdings [31–33]. The forward-looking approach under IFRS 9 was designed to improve the timeliness and adequacy of LLPs and to mitigate their procyclical effects [34]. However, the actual effects of IFRS 9 in practice remain insufficiently understood. This highlights the need for further empirical research to better evaluate the relationship between IFRS 9 adoption and procyclicality.

### The Chinese political system and the timeliness of loan loss provision under IFRS 9

Chinese political system characterized by high influence of the local leaders over the local economic conditions [35]. In this research, the local means province, and the local leader refers to the secretary of the provincial Party’s committee. China’s administrative regions are divided into five levels: the first level is the national level, i.e., the central level. The remaining four levels are collectively referred to as local levels, including the province, the city, the county, and the township [36]. The Party committee is established at each level and the secretary of the committee is the top leader at the corresponding level [37]. Since the province is the highest local administrative unit, the secretary of the provincial Party’s committee is therefore the most powerful leader at the local level.

The influence of local leaders is determined by two factors: the one-party system and high autonomy in local economic affairs. The Chinese Communist Party (CCP) is the only ruling Party recognized by the Chinese Constitution [38]. The centralization of political power endows the CCP with exceptional influence over the entire spectrum of governance, including economics. The hierarchical structure extends from the central government to the local levels, guaranteeing the seamless execution of the CCP’s policies and directives without substantial opposition or dissent. The discretion in local economic affairs underscores the profound influence of the local leaders over the economic decisions and developments in their respective regions. They are not only tailoring central directives but also responsible for creating policies to address the unique affairs in their region. [39]. As such, local leaders’ vision, priorities, and strategies have a direct and lasting impact on economic policies.

When local leaders are replaced, a shift in ideologies, economy, and strategies is usually introduced [40]. With the new leaders assuming office, their unique perspectives and priorities may diverge from their predecessors. For instance, the incoming leader might steer the local economy towards eco-friendly practices, even at the expense of rapid economic growth [41]. Such a change in leadership can precipitate alterations in local regulations and incentives, potentially affecting the behaviour of businesses, industries, and the overall economic trajectory, increasing the uncertainty of future economic conditions. In addition, the economy is an inherently intricate and interconnected system. It is sensitive to policy changes. The repercussions may extend far beyond the initial decisions [42]. This dynamism sets a cascade of effects throughout the economy, akin to a series of dominoes falling. Economic cause-and-effect intricacies make it challenging to predict with precision policies and exacerbate uncertainties in the economic landscape. Therefore, the unpredictability introduced by these shifts in the local leadership can breed economic uncertainty and render the task of foreseeing future economic outcomes [43].

In the context of IFRS 9, the interplay between ECL estimation and macroeconomic data takes a critical role in the accurate assessment of future financial risks. The effectiveness of ECL models hinges on the quality and precision of future macroeconomic information used in their calculation. Accurate forecasting of macroeconomic data enables the ECL model to reliably predict and pre-emptively identify potential future losses [3]. If the forthcoming macroeconomic landscape, especially the economic downturns, cannot be predicted with sufficient accuracy, the ECL may not be duly recognized, and the provision may be delayed. Such delayed provisions undermine the ECL model’s intended purpose of mitigating procyclicality.

In summary, the susceptibility of the ECL model to the inaccuracy of future economic indicators impacts its countercyclical efficacy. Local leaders’ turnover leads to changes in the economic policies and increases the economic uncertainty, resulting in less precise predictions of the future economic conditions. In an environment where economic forecasting is imprecise or lagging, the ability of the ECL model to fulfill its role in earlier recognizing credit risks is compromised. Therefore, the local leaders’ turnover may be a factor that affects the countercyclical effect of IFRS 9.

### Literature review

Recent empirical studies have begun to provide systematic evidence on the consequences of ECL adoption. Prisco et al. (2025) use a large EU dataset of 16,740 bank-year observations between 2012 and 2023, report that ECL adoption is associated with reduced provisioning procyclicality, but also with heightened capital management activities [44]. Their difference-in-differences analysis reveals a nuanced effect: while forward-looking provisioning mitigates the “too little, too late” problem, it simultaneously expands managerial discretion, thereby enabling earnings and capital management. Notably, auditor specialization and strong regulatory quality temper these opportunistic tendencies, suggesting that institutional oversight remains a crucial moderating factor.

In the Chinese context, Li et al. (2025) extend the analysis by introducing the concept of Delayed Expected Loan Loss Recognition (DELR), a phenomenon whereby banks postpone the recognition of expected losses despite forward-looking requirements. Using quarterly data from 16 Chinese banks over 2011–2023, they find that DELR exacerbates LLP procyclicality, and that ECL reform-after controlling for discretionary management motives-significantly reduces both DELR and its amplification of procyclicality [45]. This indicates that while ECL can address timing distortions in provisioning, its effectiveness depends on curbing both managerial discretion and institutional inertia.

Studies on the relationship between accounting standards and procyclicality have primarily focused on the procyclical effects of IAS 39. Several researchers [11,12,30,46,47] analyzed the mechanisms through which IAS 39 facilitated procyclicality. They argued that the Incurred Credit Loss (ICL) model only allowed provisions to be recognized when there was clear evidence of impairment, thereby restricting the recognition of future expected losses. This approach delays recognizing provisions and exhibits a high degree of procyclicality, which fueled the financial crisis. Other researchers [48,49] investigated the procyclicality of loan loss provision under IAS 39 through an experimental approach. Their results also supported the idea that IAS 39 has a procyclical nature. For example, Sparta and Trinova (2020) investigated the procyclical effect of IAS 39 in Indonesian banks during the period of 2008–2017 [49]. The results indicated that the banks’ loan loss provisions were negatively correlated with economic growth, signaling that the loan loss provisions of the banks tend to be procyclical. Overall, these studies generally indicated that IAS 39 has a high degree of procyclicality, and the reason lies in the ICL model.

Since the mandatory adoption of IFRS 9, a substantial body of studies has been conducted to investigate the effects of the new standard, and most of them have focused on banks. One major stream of studies investigates the most direct and immediate effects of IFRS 9, i.e., how to practice the new standard [14,16,29,50–54]. Another stream of studies investigates its effects on financial reporting [55–61]. Additional studies have explored the effects of IFRS 9 on banks’ financial ratios [33,62,63] and lending behavior [64–66].

These studies provided valuable methodologies for implementing IFRS 9 and addressed several practical issues, such as estimating probability of default (PD) and loss given default (LGD) [14,50]. They also documented both positive and negative consequences of the new standard. On the positive side, IFRS 9 improved the timeliness of loan loss provisions [55,56], enhanced the transparency and comparability of accounting information [57,58], and increased the accuracy of financial instrument classification [59]. On the negative side, IFRS 9 encouraged opportunistic behavior [60,61] and increased the complexity of accounting information [14,67].

By contrast, relatively little attention has been paid to the broader economic effects of IFRS 9. Only a few studies [22,23] have examined the relationship between IFRS 9 adoption and procyclicality. Pastiranova and Witzany (2022), for example, investigated the effects of expected credit loss provisions across the economic cycle using a sample of 28 European Union member countries from the first quarter of 2015 to the third quarter of 2020 [22]. Their study captured the fluctuations in the economic cycle during the COVID-19 downturn. Using panel regressions, they found that IFRS 9 had a procyclical effect—contrary to the countercyclical effect that the standard was intended to produce. They argued that the conclusions regarding the procyclicality of LLPs under IFRS 9 may depend on the specific models chosen by banks and the assumptions used to incorporate forward-looking information. Accordingly, they suggested that supervisory and regulatory authorities should focus on improving the quality and predictive capacity of ECL models to help mitigate potential sources of procyclicality.

A recently published article by Buesa et al. (2023) compared the procyclicality under three different accounting standards, i.e., the IFRS 9, IAS 39, and the US GAAP [23]. Their result indicates that IFRS 9 is less procyclical than IAS 39, but it remains more procyclical than US GAAP. They argue that the difference between IFRS 9 and US GAAP comes from the difference in the regulations related to the expected credit loss under these two standards. In the initial recognition of the financial instruments, IFRS 9 accounts for the one-year expected credit loss. However, in the case of US GAAP, it accounts for the expected credit loss over the life of the financial instruments. The accounting method under US GAAP comes at the cost of a large increase in provisions that occur primarily during longer contractionary phases.

The mixed results reported by Pastiranova and Witzany (2022) and Buesa et al. (2023) suggest that the impact of IFRS 9 on procyclicality may vary across countries [22]. However, the reasons underlying these differences remain underexplored. Institutional factors—such as the political environment—are known to interact with accounting standards and influence the outcomes of IFRS adoption [55,68]. This highlights the importance of considering such factors in the implementation of IFRS 9. Yet, the existing literature has largely overlooked the role of institutional contexts. This gap suggests the need for further research to better understand how IFRS 9 interacts with institutional factors and how these interactions shape its impact on procyclicality.

### Theoretical framework and hypothesis development

#### The financial accelerator theory.

The Financial Accelerator theory was first introduced by Bernanke, Gertler, and Gilchrist (1994). They defined the financial accelerator as “the amplification of initial shocks brought about by changes in credit market conditions” [69]. In essence, the theory explains how adverse economic shocks can be magnified when credit market conditions deteriorate. Earlier, Bernanke et al. (1986) argued that borrowers engage in investment and productive activities primarily by relying on bank loans [69]. Because of information asymmetry, e.g., banks’ limited knowledge of borrowers’ investment and production prospects, banks typically require collateral as evidence of repayment capacity. The cost of borrowing is closely linked to the value of collateral, which mitigates the risk of default. There is an inverse relationship between collateral value and borrowing costs: the higher the collateral value, the lower the borrowing costs. Collateral values are generally tied to borrowers’ net worth, defined as the sum of liquid assets plus the collateral value of illiquid assets minus outstanding obligations [70]. Thus, when borrowers’ net worth is high, they can pledge more assets, leading to lower borrowing costs; conversely, when net worth is low, borrowing becomes more expensive.

During economic downturns, asset prices typically fall, reducing borrowers’ net worth and eroding their balance sheets [70]. As net worth declines, borrowing costs rise and borrowing capacity contracts, limiting borrowers’ ability to invest and produce. The resulting reduction in economic activity further depresses asset prices, creating a feedback loop of declining asset values, weaker balance sheets, tighter financing conditions, and reduced economic activity. This self-reinforcing cycle constitutes the financial accelerator effect.

Fundamentally, the financial accelerator effect and the procyclical effect share the same mechanism. The theory highlights the interaction between financial markets and the business cycle, showing how borrowing costs fluctuate in tandem with economic conditions. Since credit risk moves with the cycle, the financial accelerator effect parallels the concept of procyclicality, which also denotes the correlation between risk and the economic cycle. In financial statements, credit risk is reflected in loan loss provisions (LLPs). When LLPs fluctuate synchronously with the cycle, the procyclical effect emerges. Thus, the financial accelerator theory provides a useful framework for understanding the procyclicality of LLPs.

Procyclicality stems from the synchronicity between fluctuations in LLPs and the economic cycle. Reducing this synchronicity is therefore key to mitigating procyclicality. Under IAS 39, LLPs could only be recognized after losses had occurred, causing LLPs to move in lockstep with the cycle and reinforcing procyclicality [71,72]. By contrast, IFRS 9 was designed to anticipate credit losses and thereby reduce this synchronicity. Conceptually, the expected credit loss (ECL) model does not change the total amount of credit losses recognized over a downturn; rather, it alters their timing. A significant portion of losses is recognized at the onset of, or even before, an economic downturn. Banks are thus required to estimate provisions based on both current and expected future losses. This forward-looking approach limits the additional provisioning required at the moment of default, thereby smoothing cyclical volatility and easing capital pressure [3]. In this way, IFRS 9 is expected to mitigate procyclicality [73].

Based on the analysis above, Hypothesis 1 was put forward.

Hypothesis 1: IFRS 9 adoption weakens the procyclicality.

#### Institutional theory.

The Institutional Theory is a sociological and organizational theory that examines how institutions shape and influence individuals, organizations, and societies [74]. It posits that external factors such as culture, regulations, policies, and other institutional elements influence organizational behavior [75]. In the accounting field, institutional theory helps explain why firms adopt specific accounting practices, conform to certain accounting standards, and how they interact with their institutional environment [76].

Accounting standards are important institutional factors, as they shape organizations’ accounting practices [77,78]. However, accounting standards do not operate in isolation; they interact with other institutional factors, such as the economic and political environment, which affect the outcomes of adopting new standards [75]. Specifically, under IFRS 9, expectations of future economic conditions are a key input to the expected credit loss (ECL) model, determining the accuracy of ECL and loan loss provision (LLP) estimates. In China, local economic conditions are strongly influenced by local leaders, whose policies, such as tax, monetary, and fiscal measures, significantly affect corporate financing, investment behavior, industrial development, and overall regional economic growth [79].

When local leadership turnover occurs, economic policy inconsistencies often arise, as new leaders typically introduce new policies. This results in policy uncertainty, making future economic conditions less predictable [43]. Since the ECL model relies on accurate forecasts of future economic conditions, reduced predictability leads to less reliable ECL estimates. If future economic downturns cannot be foreseen, banks may become overly optimistic and underestimate default probabilities. Consequently, LLPs may be delayed, resembling provisions under IAS 39, thereby producing a procyclical effect. Therefore, local leaders’ turnover may hinder the mitigative effect of IFRS 9 adoption on procyclicality.

Based on the analysis above, Hypothesis 2 was put forward.

Hypothesis 2: The turnover of the local leaders weakens the mitigative effect of IFRS 9 adoption on the procyclicality.

### Research methodology

#### Research design.

This research is empirical and quantitative in nature. As the primary objective is to investigate the procyclicality of loan loss provisions (LLP), the research tests the relationship between LLP and the economic cycle, proxied by GDP growth. A significant negative relationship between LLP and GDP growth indicates the presence of procyclicality. This approach has been widely used in prior research [80–83] to examine procyclicality, and GDP growth is a commonly accepted indicator of the economic cycle [83–85].

Since IFRS 9 primarily affects banks due to their extensive holdings of financial instruments, the research focuses exclusively on the banking sector. The sample consists of local banks that operate mainly within the provinces where they are located. Accordingly, the GDP growth rate used is the provincial (local-level) GDP growth rate. Procyclicality is thus represented by the correlation between local banks’ LLP and local GDP growth.

The research period spans 2019–2022, with 2019–2020 representing the IAS 39 era and 2021–2022 representing the IFRS 9 era. The research evaluates the impact of IFRS 9 by comparing the procyclical effect of LLP before and after its implementation. It further investigates the influence of local leaders’ turnover, earnings management, and the legal environment by introducing corresponding representative variables.

Following the methodology of Pastiranova and Witzany (2022), this research also incorporates control variables commonly applied in previous literature [e.g., 8,80–82,86–88] on procyclicality. These include the nonperforming loan ratio, Tier 1 capital ratio, bank size (proxied by the logarithm of total assets), loan growth rate, the ratio of loan loss allowance to total loans, the ratio of loans to total assets, and earnings before tax and provisions. The banks’ data and GDP are collected from the WIND database. The variables used in this research, along with their definitions and measurements, are presented in Table 1.

**Table 1:** Definitions of variables.

Variables	Definition	Formula
LLP	Loan loss provision rate	LLP_it_ = Loan Loss Provision_it_ ÷Total Loans_it_ × 100%
LLP (−1)	Loan loss provision rate of prior year	
LLP (−2)	Loan loss provision rate of the year before prior year	
GDP	Current real gross domestic product growth rate	GDP_t_ = (GDP_t_ - GDP_t−1_)÷GDP_t−1_ × 100%
NGDP	Current nominal GDP growth rate	Nominal GDP_t_ = (nominal GDP_t_ – nominal GDP_t−1_) ÷ nominal GDP_t−1_ × 100%
NPL	Nonperforming loan rate at the beginning of the year	NPL_it_ = nonperforming loan_it_ ÷ total loan_it-1_ × 100%
CAP	Tier 1 capital ratio at the beginning of the year	CAP_it_ = Tier 1 capital ÷ Weighted asset risk ×100%
SIZE	The size of the bank which proxied by the logarithm of the total assets	SIZE = log (total assets)
LOAN	Loan growth rate	LOAN_it_ = (total loan_it_ – total loan_it-1_) ÷ total loan_it-1 _× 100%
LLA	The ratio of the loan loss allowance to the total loan at the beginning of the year	LLA_it_ = loan loss allowance_it_ ÷ total loan_it _× 100%
LA	The ratio of loan to total asset	LA_it_ = total loan_it_ ÷ total assets_it _× 100%
EBTP	The ratio of earnings before tax and LLP to the total asset	EBTP_it_ = earnings before tax and loan loss provision_it_ ÷ total assets_it _× 100%
TOLL	Turnover of the local leaders, it is a dummy variable which equals 1 if the local leader turnover and the 0 otherwise	
u	Bank fixed effect	
v	Time fixed effect	
e	Error term	

### Sample and data collection

In this research, we measure procyclicality through the relationship between loan loss provisions (LLP) and GDP growth rates. To ensure sufficient variation and a meaningful number of observations, the sample must consist of regionally operated banks, with GDP growth measured at the regional level (e.g., province). If nationwide banks were included, their LLP would need to be regressed against the national GDP growth rate. However, since the national GDP growth rate is a single figure, it would provide insufficient variation in the explanatory variable and render the analysis statistically infeasible. Therefore, to align banks’ LLP with the corresponding economic cycle, the sample is limited to regionally operated banks whose business activities are confined to a single province. This geographic alignment ensures that regional GDP growth rates appropriately capture the economic environment faced by each bank, thereby enabling more accurate estimation of procyclicality.

The objective of this research is to investigate the relationship between local banks’ LLP and local GDP growth. Accordingly, the sample includes only local commercial banks in China. Local banks in China can be classified into four categories based on their listing status: (1) banks listed both domestically and abroad (adopting IFRS 9 on January 1, 2018), (2) banks listed only abroad (adopting IFRS 9 on January 1, 2018), (3) banks listed only domestically (adopting IFRS 9 on January 1, 2019), and (4) unlisted banks (adopting IFRS 9 on January 1, 2021). The total population of listed local banks is only 18. Given the small sample size and the staggered IFRS 9 adoption timelines, these listed banks are excluded from the research. Ultimately, the sample consists of 175 unlisted local banks.

For unlisted banks in China, IFRS 9 became mandatory on January 1, 2021. To capture the post-adoption effects of IFRS 9, the research period is restricted to 2019–2022, covering the years immediately before and after the accounting standard change. The years 2019–2020 represent the IAS 39 period, while 2021–2022 represent the IFRS 9 period. Given the relatively short post-adoption window and data availability, this research focuses on the short-term impact of IFRS 9 on the procyclicality of LLP. By examining the immediate years following implementation, this research aims to identify how banks’ provisioning behavior adjusted in response to the expected credit loss (ECL) model. In addition, data on local leaders’ turnover (measured as the secretary of the local Party committee) are hand-collected from local government websites. The local governments’ websites are listed at the end of the paper, see Supporting Information S1 Fig.

### Research equations

To examine the impact of IFRS 9 on the procyclicality of loan loss provisions (LLP), this research employs a baseline regression model. Following Pastiranova and Witzany (2022) [22], we test the relationship between LLP and GDP growth under IAS 39 and IFRS 9, respectively. The research period is divided into pre-adoption and post-adoption phases of IFRS 9, allowing for a direct comparison of LLP’s responsiveness to economic cycles across the two regulatory regimes. This approach provides insight into whether the introduction of IFRS 9 mitigates procyclicality.

In the model, LLP serves as the dependent variable, while GDP growth is the independent variable. The specification captures how macroeconomic conditions influence banks’ LLP. Specifically, if LLP decreases during periods of economic expansion and increases during downturns, it reflects procyclical behavior. To control for unobserved, time-invariant heterogeneity across banks and for common shocks across years, the model incorporates two-way fixed effects. In addition, a set of bank-level control variables is included to ensure the robustness of the empirical findings. This design enables a nuanced analysis of the interaction between the business cycle and banks’ risk management practices.

The specific model is formulated as follows:


$$\begin{array}{c} {LLP}_{it}(IAS\text{3}\text{9})=\;\alpha_{\text{0}}+\alpha_{\text{1}}{GDP}_{it}+\alpha_{\text{2}}{LLP}_{it-\text{1}}+\alpha_{\text{3}}{LLP}_{it-\text{2}}\\ \;\;\;\;\;\;+\;\alpha_{\text{4}}{NPL}_{it-\text{1}}+\alpha_{\text{5}}{CAP}_{it-\text{1}}+\alpha_{\text{6}}{SIZE}_{it}+\alpha_{\text{7}}{LOAN}_{it}\\ \;\;\;\;\;\;+\;\alpha_{\text{8}}{LLA}_{it-\text{1}}+\alpha_{\text{9}}{LA}_{it}+\alpha_{\text{1}\text{0}}{EBTP}_{it}+u+v+\varepsilon_{it} \end{array}$$ (1)

$$\begin{array}{c} {LLP}_{it}(IFRS\text{9})=\;\beta_{\text{0}}+\beta_{\text{1}}{GDP}_{it}+\beta_{\text{2}}{LLP}_{it-\text{1}}+\beta_{\text{3}}{LLP}_{it-\text{2}}+\beta_{\text{4}}{NPL}_{it-\text{1}}\\ \;\;\;\;\;\;+\beta_{\text{5}}{CAP}_{it-\text{1}}+\beta_{\text{6}}{SIZE}_{it}+\beta_{\text{7}}{LOAN}_{it}+\beta_{\text{8}}{LLA}_{it-\text{1}}\\ \;\;\;\;\;\;+\;\beta_{\text{9}}{LA}_{it}+\beta_{\text{1}\text{0}}{EBTP}_{it}+u+v+\varepsilon_{it} \end{array}$$ (2)

Where:

LLP = loan loss provision rate to the total loan

GDP = real GDP growth rate

NPL = nonperforming loan rate to the total loan

CAP = tier 1 capital adequacy ratio

SIZE = the logarithm of total assets

LOAN = loan growth rate

LLA = loan loss allowance rate to the total loan

LA = loan rate to the total asset

EBTP = earnings before tax and provision rate to the total asset

u = bank fixed effect

v = time fixed effect

e = error term

The coefficients α1 and β1 capture the degree of procyclicality under IFRS 9 and IAS 39, respectively. If these coefficients are negative and statistically significant, this implies that current LLP is negatively associated with contemporaneous GDP growth, indicating a procyclical effect—which is generally undesirable.

To examine the influence of local leaders’ turnover on the procyclicality of LLP, we introduce the dummy variable TOLL, which equals one in years of local leader turnover and zero otherwise. This variable allows us to test how political turnover affects the relationship between LLP and GDP growth. Specifically, the interaction term (GDP × TOLL) captures whether local leader turnover moderates the responsiveness of LLP to economic conditions. Put differently, we test whether LLP’s procyclicality changes during periods of political uncertainty. A negative and significant coefficient on the interaction term would indicate that during turnover periods, LLP’s negative sensitivity to GDP growth is amplified, implying that political uncertainty strengthens procyclicality.

The model is specified as follows:


$$\begin{array}{c} {LLP}_{it}(IAS\text{3}\text{9})=\;\gamma_{\text{0}}+\gamma_{\text{1}}{GDP}_{it}\times TOLL+\gamma_{\text{2}}{GDP}_{it}+\gamma_{\text{3}}TOLL\\ \;\;\;\;\;\;+\;\gamma_{\text{4}}{LLP}_{it-\text{1}}+\gamma_{\text{5}}{LLP}_{it-\text{2}}+\gamma_{\text{6}}{NPL}_{it-\text{1}}+\gamma_{\text{7}}{CAP}_{it-\text{1}}\\ \;\;\;\;\;\;+\;\gamma_{\text{8}}{SIZE}_{it}+\gamma_{\text{9}}{LOAN}_{it}+\gamma_{\text{1}\text{0}}{LLA}_{it-\text{1}}+\gamma_{\text{1}\text{1}}{LA}_{it-\text{1}}\\ \;\;\;\;\;\;+\;\gamma_{\text{1}\text{2}}{EBTP}_{it}+u+v+\varepsilon_{it} \end{array}$$ (3)

$$\begin{array}{c} {LLP}_{it}(IAS\text{3}\text{9})=\;\delta_{\text{0}}+\delta_{\text{1}}{GDP}_{it}\times TOLL+\delta_{\text{2}}{GDP}_{it}+\delta_{\text{3}}TOLL\\ \;\;\;\;\;\;+\;\delta_{\text{4}}{LLP}_{it-\text{1}}+\delta_{\text{5}}{LLP}_{it-\text{2}}+\delta_{\text{6}}{NPL}_{it-\text{1}}+\delta_{\text{7}}{CAP}_{it-\text{1}}\\ \;\;\;\;\;\;+\;\delta_{\text{8}}{SIZE}_{it}+\delta_{\text{9}}{LOAN}_{it}+\delta_{\text{1}\text{0}}{LLA}_{it-\text{1}}+\delta_{\text{1}\text{1}}{LA}_{it-\text{1}}\\ \;\;\;\;\;\;+\;\delta_{\text{1}\text{2}}{EBTP}_{it}+u+v+\varepsilon_{it} \end{array}$$ (4)

Where:

TOLL = turnover of the local leader, a dummy variable, which equals 1 if the leaders’ turnover happened in the current year and 0 otherwise

The definitions of the other variables remain the same as in equation (1). The coefficients γ1 and δ1 capture the effect of TOLL on the relationship between LLP and GDP growth during the IAS 39 period and the IFRS 9 period, respectively. If γ1 and δ1 are negative and statistically significant, this indicates that TOLL amplifies the procyclical effect of LLP.  Table 2 presents the descriptive statistics and Table 3 presents the correlation analysis.

**Table 2:** Descriptive Statistics.

Variable	Obs	Mean	Std. Dev.	Min	Max
LLP	700	1.22	.609	.003	3.736
LOAN	700	17.29	8.196	.112	88.236
SIZE	700	10.788	.482	9.766	12.037
LLA	700	4.433	1.632	1.416	11.328
NPL	700	1.745	1.072	.452	13.182
LLP (−1)	700	1.386	.695	.003	5.076
LLP (−2)	700	1.49	.77	.003	6
CAP	700	11.52	2.332	.74	18.517
NPL	700	1.818	1.005	.49	7.321
LA	700	57.385	8.023	29.019	80.461
EBTP	700	1.595	.504	.085	3.953
GDP	700	5.416	2.185	1.1	9.3
NGDP	700	7.585	4.166	.711	28.229
TOLL	700	.407	.492	0	1

**Table 3:** Correlation analysis.

	LLP	GDP	LOAN	SIZE	LLA	NPL	LLP (−1)	LLP (−2)	CAP	LA	EBTP	TOLL
LLP	1											
GDP	0.0590	1										
	0.122											
LOAN	−0.0470	0.0260	1									
	0.211	0.495										
SIZE	0.0340	−0.102***	0.097***	1								
	0.370	0.00670	0.00990									
LLA	0.0480	0.082**	0.0460	−0.540***	1							
	0.202	0.0308	0.220	0								
NPL	0.205***	0.0270	−0.225***	−0.222***	0.193***	1						
	0	0.483	0	0	0							
LLP (−1)	0.711***	0.169***	0.0180	−0.116***	0.292***	0.289***	1					
	0	0	0.625	0.00220	0	0						
LLP (−2)	0.475***	0.00800	0.070*	−0.243***	0.381***	0.235***	0.693***	1				
	0	0.841	0.0651	0	0	0	0					
CAP	−0.069*	0.0350	−0.0150	−0.164***	0.211***	−0.248***	−0.0220	0.0310	1			
	0.0700	0.357	0.692	0	0	0	0.564	0.408				
LA	−0.085**	−0.00200	0.0180	−0.214***	0.109***	−0.239***	−0.105***	−0.0580	0.126***	1		
	0.0248	0.967	0.627	0	0.00400	0	0.00540	0.122	0.000800			
EBTP	0.461***	0.149***	−0.0140	−0.247***	0.296***	−0.234***	0.350***	0.237***	0.334***	0.308***	1	
	0	0.000100	0.706	0	0	0	0	0	0	0		
TOLL	−0.109***	−0.382***	0.0140	−0.0280	−0.0190	−0.082**	−0.135***	0.00900	−0.076**	0.138***	−0.145***	1
	0.00390	0	0.708	0.465	0.608	0.0307	0.000400	0.810	0.0457	0.000200	0.000100	

*t* statistics in parentheses

* *p* < 0.1, ** *p* < 0.05, *** *p* < 0.01

### Empirical results

According to the regression results shown in Table 4, the coefficient of GDP (α1) in the IAS 39 group is −0.074, negative and statistically significant. This indicates a negative relationship between LLP and GDP growth during the IAS 39 period: a one-percentage-point decrease in GDP growth is associated with a 0.074-percentage-point increase in LLP, demonstrating the procyclical nature of provisions under IAS 39. In contrast, the coefficient of GDP in the IFRS 9 group (β1) is −0.006 and not statistically significant, suggesting that the procyclical effect of provisions under IFRS 9 has been mitigated, supporting our first hypothesis.

**Table 4:** Regression results.

	(1)	(2)	(3)	(4)
	LLP(IAS39)	LLP(IFRS9)	LLP(IAS39)	LLP(IFRS9)
GDP	−0.074^**^	−0.006	−0.098^***^	0.002
	(−2.01)	(−0.28)	(−2.63)	(0.09)
				
TOLL*GDP			−0.045	−0.030^*^
			(−0.70)	(−1.89)
				
TOLL			0.027	0.179^*^
			(0.11)	(1.81)
				
LOAN	−0.003	0.001	−0.001	0.002
	(−0.78)	(0.40)	(−0.42)	(0.59)
				
SIZE	−0.381	−3.548^***^	−0.193	−3.662^***^
	(−0.51)	(−3.62)	(−0.26)	(−3.64)
				
LLA	−0.105^*^	−0.089^***^	−0.108^*^	−0.084^***^
	(−1.81)	(−3.00)	(−1.90)	(−2.85)
				
NPL	0.159^**^	−0.044	0.156^**^	−0.057
	(2.29)	(−0.78)	(2.27)	(−1.00)
				
LLP (−1)	0.039	−0.046	0.020	−0.047
	(0.81)	(−0.88)	(0.40)	(−0.90)
				
LLP (−2)	−0.042	−0.085^*^	−0.058	−0.093^*^
	(−0.88)	(−1.74)	(−1.23)	(−1.92)
				
CAP	−0.019	−0.036	−0.020	−0.031
	(−0.68)	(−1.65)	(−0.74)	(−1.39)
				
LA	−0.036^***^	−0.050^***^	−0.037^***^	−0.052^***^
	(−3.05)	(−5.42)	(−3.17)	(−5.67)
				
EBTP	1.301^***^	0.963^***^	1.272^***^	0.971^***^
	(10.11)	(8.88)	(9.94)	(8.99)
				
_cons	6.045	42.117^***^	4.363	43.381^***^
	(0.71)	(3.84)	(0.52)	(3.87)
N	350.000	350.000	350.000	350.000
r2_a	0.820	0.888	0.825	0.889
F	12.095	14.141	10.927	12.199
Bankcode	Yes	Yes	Yes	Yes
Year	Yes	Yes	Yes	Yes

*t* statistics in parentheses

* *p* < 0.1, ** *p* < 0.05, *** *p* < 0.01

The mitigation of procyclicality under IFRS 9 reflects the countercyclical design of the expected credit loss (ECL) model. Under this model, banks incorporate forward-looking information and recognize provisions earlier, even before defaults occur. This mechanism smooths LLP across different stages of the economic cycle, promoting a more stable capital buffer. Furthermore, IFRS 9 allows banks to use internal credit risk models, which can better anticipate future economic conditions and inform provisioning decisions, reducing reliance on reactive practices. These findings align with prior studies [89] indicating that IFRS 9 adoption mitigates procyclicality.

Regarding the interaction term TOLL × GDP, the coefficient in the IAS 39 group (γ1) is 0.045 and not statistically significant, indicating that local leader turnover had no significant effect on the relationship between LLP and GDP growth under IAS 39. In the IFRS 9 group, the coefficient (δ1) is −0.030 and statistically significant, suggesting that turnover of local leaders moderates the relationship between LLP and GDP growth. Specifically, political turnover appears to weaken the mitigative effect of IFRS 9 on procyclicality, supporting our second hypothesis.

These results imply that the countercyclical effect of IFRS 9 is hindered by local leader turnover. Changes in leadership increase regulatory and political uncertainty, as new leaders often introduce different policies and priorities. This uncertainty makes future economic conditions less predictable and reduces the effectiveness of forward-looking provisioning, delaying LLP recognition and partially reintroducing procyclicality. Moreover, under uncertain conditions, banks have greater discretion in provisioning, which may further exacerbate cyclical fluctuations. These findings are consistent with prior studies [90,91] suggesting that local leader turnover increases political and economic uncertainty and fosters earnings management by banks.

## Discussion

The empirical results provide clear evidence that LLP under IAS 39 exhibits procyclicality, and the adoption of IFRS 9 mitigates this procyclical effect. Under IAS 39, the negative and statistically significant coefficient of GDP (−0.074) indicates that LLP increases with GDP growth decline. This finding aligns with prior studies that argue the incurred credit loss (ICL) model amplifies economic fluctuations by delaying recognition of provision and thereby reinforcing both booms and busts. Under this reactive framework, banks reduced provisions during economic expansions—boosting profits and lending—and sharply increased provisions in recessions, constraining credit supply and exacerbating downturns.

In contrast, during the IFRS 9 period, the coefficient of GDP (−0.006) was statistically insignificant, suggesting that the ECL model largely decoupled LLP from short-term economic fluctuations. This supports our first hypothesis and indicates that IFRS 9’s forward-looking approach—requiring earlier recognition of expected losses—helped smooth provisioning over the economic cycle. By incorporating macroeconomic forecasts and allowing banks to design internal credit risk models, IFRS 9 encouraged more anticipatory provisioning, thereby reducing reliance on purely backward-looking indicators. This shift is consistent with previous research [87] showing that the ECL model can serve as a countercyclical buffer, stabilizing bank capital and credit supply across economic conditions.

However, the results also reveal that the benefits of IFRS 9 are not uniform across all institutional contexts. The significant negative coefficient for TOLLGDP (−0.030) in the IFRS 9 period indicates that local leaders’ turnover weakens the standard’s mitigative effect on procyclicality, supporting our second hypothesis. In contrast, under IAS 39, the coefficient for TOLLGDP was insignificant, suggesting that political turnover did not materially alter procyclicality in the ICL framework. This difference likely reflects the heightened sensitivity of the ECL model to forward-looking economic inputs: when political turnover increases policy and regulatory uncertainty, banks face greater difficulty in forecasting future economic conditions. This uncertainty reduces the accuracy of ECL estimates, prompting banks to revert to more discretionary or delayed provisioning practices, which reintroduces elements of procyclicality.

These findings are consistent with prior literature [90,91] emphasizing that political turnover in China generates uncertainty in local economic policy direction, investment climate, and regulatory enforcement. This uncertainty can incentivize earnings management behaviors by banks, as they adjust provisioning to smooth reported results in the face of unpredictable policy environments. Importantly, our results suggest that while IFRS 9 improves the cyclical stability of provisioning, its effectiveness is contingent upon a stable institutional and political environment. Without such stability, the countercyclical potential of forward-looking provisioning models may be compromised.

Overall, the discussion highlights two key implications. First, the procyclicality cannot be fully addressed by IFRS 9 alone. Institutional factors such as leadership and policy stability also play a crucial role in shaping the consequences of IFRS 9’s adoption. Second, policymakers and standard setters who aim to maximize the countercyclical effect of IFRS 9 may complement accounting reforms with institutional measures that constrain political and economic uncertainty. For instance, enhancing policy continuity during the leadership transitions, improving the reliability of macroeconomic forecasting, and strengthening supervisory oversight to limit discretionary provisioning practices under uncertain conditions.

### Robustness test

In this section, three robustness tests are conducted to improve the credibility of the empirical results. First, the LLP rate relative to total loans is replaced with the LLP rate relative to total assets. Second, following Olszak et al. (2018), the nominal GDP growth rate (NGDP) is used to replace the real GDP growth rate, and the relationship between NGDP and LLP is examined. Third, following Olszak et al. (2017), the lagged variable LLP(−2) is excluded to examine whether the results change significantly. The results of these robustness tests are presented in Table 5. Overall, the key coefficients and their statistical significance remain largely unchanged, indicating that our findings are robust.

**Table 5:** Robustness Check.

	(1)	(2)	(3)	(4)	(5)	(6)	(7)	(8)	(9)	(10)	(11)	(12)
	LLPTA (IAS39)	LLPTA (IFRS9)	LLPTA (IAS39)	LLPTA (IFRS9)	LLP (IAS39)	LLP (IFRS9)	LLP (IAS39)	LLP (IFRS9)	LLP (IAS39)	LLP (IFRS9)	LLP (IAS39)	LLP (IFRS9)
GDP	−0.041^**^	−0.004	−0.057^***^	0.001					−0.075^**^	−0.002	−0.101^***^	0.005
	(−2.04)	(−0.29)	(−2.78)	(0.09)					(−2.07)	(−0.10)	(−2.73)	(0.21)
												
NGDP					−0.044^**^	−0.001	−0.067^***^	0.006				
					(−2.10)	(−0.11)	(−3.00)	(0.38)				
												
TOLL*GDP			−0.027	−0.019^**^							−0.040	−0.028^*^
			(−0.77)	(−2.02)							(−0.63)	(−1.71)
												
TOLL*NGDP							−0.006	−0.014^*^				
							(−0.12)	(−1.93)				
												
TOLL			0.010	0.112^*^			−0.146	0.150^*^			0.012	0.162
			(0.07)	(1.96)			(−0.70)	(1.81)			(0.05)	(1.64)
												
LOAN	−0.001	0.001	−0.001	0.001	−0.003	0.001	−0.001	0.001	−0.003	0.001	−0.002	0.002
	(−0.75)	(0.50)	(−0.32)	(0.70)	(−0.82)	(0.39)	(−0.30)	(0.50)	(−0.95)	(0.42)	(−0.56)	(0.60)
												
SIZE	−0.275	−1.942^***^	−0.155	−2.027^***^	−0.401	−3.512^***^	−0.231	−3.644^***^	−0.142	−3.518^***^	0.050	−3.620^***^
	(−0.66)	(−3.40)	(−0.38)	(−3.47)	(−0.53)	(−3.52)	(−0.31)	(−3.58)	(−0.20)	(−3.57)	(0.07)	(−3.58)
												
LLA	−0.050	−0.045^**^	−0.052^*^	−0.042^**^	−0.112^*^	−0.089^***^	−0.118^**^	−0.089^***^	−0.088	−0.106^***^	−0.098^*^	−0.103^***^
	(−1.56)	(−2.60)	(−1.66)	(−2.44)	(−1.93)	(−3.03)	(−2.08)	(−3.02)	(−1.61)	(−3.76)	(−1.80)	(−3.68)
												
NPL	0.083^**^	−0.012	0.082^**^	−0.020	0.165^**^	−0.045	0.174^**^	−0.045	0.153^**^	−0.060	0.148^**^	−0.072
	(2.18)	(−0.38)	(2.17)	(−0.60)	(2.39)	(−0.78)	(2.55)	(−0.78)	(2.24)	(−1.11)	(2.17)	(−1.31)
												
LLP (−1)	0.031	−0.026	0.018	−0.027	0.041	−0.045	0.016	−0.049				
	(1.17)	(−0.87)	(0.69)	(−0.90)	(0.84)	(−0.85)	(0.33)	(−0.94)				
												
LLP (−2)	−0.021	−0.044	−0.032	−0.049^*^	−0.037	−0.084^*^	−0.052	−0.095^*^				
	(−0.80)	(−1.55)	(−1.21)	(−1.74)	(−0.77)	(−1.72)	(−1.10)	(−1.95)				
												
CAP	−0.006	−0.015	−0.007	−0.011	−0.014	−0.035	−0.013	−0.032	−0.016	−0.038^*^	−0.016	−0.033
	(−0.40)	(−1.17)	(−0.46)	(−0.90)	(−0.52)	(−1.63)	(−0.49)	(−1.49)	(−0.60)	(−1.72)	(−0.59)	(−1.49)
												
LA	−0.007	−0.017^***^	−0.008	−0.019^***^	−0.035^***^	−0.049^***^	−0.036^***^	−0.052^***^	−0.035^***^	−0.048^***^	−0.036^***^	−0.050^***^
	(−1.06)	(−3.20)	(−1.19)	(−3.48)	(−3.01)	(−5.41)	(−3.12)	(−5.64)	(−2.94)	(−5.26)	(−3.07)	(−5.46)
												
EBTP	0.727^***^	0.558^***^	0.708^***^	0.563^***^	1.294^***^	0.965^***^	1.250^***^	0.979^***^	1.293^***^	1.002^***^	1.262^***^	1.011^***^
	(10.23)	(8.85)	(10.09)	(8.97)	(10.06)	(8.94)	(9.81)	(9.09)	(10.08)	(9.43)	(9.89)	(9.53)
												
_cons	3.203	22.363^***^	2.128	23.301^***^	6.068	41.687^***^	4.556	43.121^***^	3.317	41.565^***^	1.557	42.681^***^
	(0.68)	(3.50)	(0.46)	(3.58)	(0.71)	(3.74)	(0.55)	(3.81)	(0.41)	(3.78)	(0.19)	(3.79)
N	350.000	350.000	350.000	350.000	350.000	350.000	350.000	350.000	350.000	350.000	350.000	350.000
r2_a	0.826	0.892	0.834	0.893	0.820	0.888	0.827	0.889	0.820	0.887	0.825	0.888
F	12.983	12.266	12.049	10.695	12.153	14.129	11.227	12.208	14.984	17.127	12.961	14.074

*t* statistics in parentheses

* *p* < 0.1, ** *p* < 0.05, *** *p* < 0.01.

## Conclusions

This research explored the impact of IFRS 9 on the procyclicality of LLP and how China’s unique political environment influences the effectiveness of IFRS 9 in mitigating the procyclicality. The Financial Accelerator Theory and Institutional Theory are adopted to form the theoretical framework and explain the mechanism of the procyclical effect as well as the interplay between IFRS 9 and local leaders’ turnover. Using the sample of 175 local commercial banks in China, this research tested the relationship between local banks’ LLP and the local GDP growth under IAS 39 and IFRS 9, respectively. The results indicated that IFRS 9 adoption mitigated the procyclicality of LLP. Further, this research examined the role of local leaders’ turnover. The results indicate that the turnover of the local leaders negatively moderates the relationship between LLP and GDP growth under IFRS 9 but does not have a significant influence on the relationship between LLP and GDP growth under IAS 39. This indicates that the local leaders’ turnover deters the mitigative effect of IFRS 9 on procyclicality.

This research expands the understanding of the economic impact of IFRS 9 beyond traditional accounting perspectives, emphasizing IFRS 9’s broader economic consequences. This research also revealed the negative effect of local leaders’ turnover on the countercyclicality of IFRS 9, providing empirical evidence for the standard setters and policymakers, aiding in the improvement of the accounting standard and the design of regulatory frameworks to mitigate the procyclical effect. Moreover, by applying the Financial Accelerator and Institutional theories, this research elucidates how accounting standards influence bank credit cycles and amplify economic fluctuations, contributing to the application of these theories within the accounting field.

Based on the research’s findings, several practical recommendations can be made for policymakers, regulators, and standard setters to strengthen the countercyclical capacity of IFRS 9 and limit procyclical risk in the banking sector. First, given that local leaders’ turnover weakens IFRS 9’s mitigative effect, mechanisms should be developed to ensure policy continuity during political transitions, such as formalizing economic development strategies, maintaining regulatory priorities, and providing clear transition guidelines to reduce uncertainty in local economic conditions-key inputs to ECL estimates. Second, to maximize the ECL model’s effectiveness, regulators should improve forward-looking data infrastructure by promoting the collection, standardization, and timely dissemination of reliable macroeconomic and sectoral forecasts, thereby reducing estimation errors and discretionary adjustments. Third, supervisory oversight of discretionary provisioning should be strengthened during periods of political or economic instability through targeted reviews of provisioning models, scenario assumptions, and credit risk forecasts to ensure consistent application of forward-looking principles. Fourth, standard setters such as the IASB should integrate institutional stability considerations into IFRS 9’s design, providing guidance for adjusting ECL methodologies in high-uncertainty contexts, including recommendations for conservative buffers or scenario weightings when forecast reliability is low. Finally, developing countries with a transitional institutional setting may require supplementary prudential measures such as dynamic provisioning rules, countercyclical capital buffers, and clear macroprudential guidance. These measures may not only guarantee IFRS 9’s countercyclical effect but also adapt its application to a specific institutional context, contributing to financial stability and sustainable economic growth.

### Limitations and future directions

Despite its contributions, this research acknowledges several limitations. First, the analysis focuses exclusively on regionally operated unlisted banks in China. While this sample choice ensures alignment between bank-level data and regional economic conditions, it may limit the generalizability of the findings to other types of banks, such as nationally operated or listed banks. Second, this research only focuses on the immediate effect of IFRS 9 on procyclicality. The research period spans only four years (2019–2022), which restricts the ability to assess the long-term impacts of IFRS 9, particularly across different phases of the economic cycle. Third, this research does not consider broader institutional or contextual factors, such as the strength of regulatory enforcement and varying market conditions. These institutional factors may interact with IFRS 9 to influence the extent to which IFRS 9 affects banks’ provisioning behavior.

Building on this research, future research could explore several avenues to deepen the understanding of the procyclicality of LLP under IFRS 9. First, expanding the sample to include multiple types of banks and multiple countries’ economic indicators. This would help assess whether there is heterogeneity in different types of banks and varying institutional settings. Second, extending the research period and involving more years. This would contribute to the assessment of the long-term effects of IFRS 9 on procyclicality over different stages of the economic cycle. Third, incorporating more institutional factors, such as regulatory enforcement and market conditions. This would provide more comprehensive insights into the mechanisms that influence banks’ provisioning practices and procyclicality.

## Supporting information

S1 Fig Data. (XLS)

S2 Fig The list of local governments’ websites. (DOCX)
