# Supplementary material for: IFRS 9 and procyclicality of loan loss provision among Chinese regional banks, the role of local leaders’ turnover
Source: PLoS One. 2025 Nov 19;20(11):e0336156. doi: 10.1371/journal.pone.0336156 (PMC12629493; doi:10.1371/journal.pone.0336156)
Supplement: S2 Fig — (DOCX) [file pone.0336156.s002.docx]

# Provinces’ Website

Hebei Province：https://www.hebei.gov.cn/

Shanxi Province：https://www.shanxi.gov.cn/

Liaoning Province：https://www.ln.gov.cn/

Jilin Province：https://www.jl.gov.cn/

Heilongjiang Province：https://www.hlj.gov.cn/

Jiangsu Province：https://www.jiangsu.gov.cn/

Zhejiang Province：https://www.zj.gov.cn/

Anhui Province：https://www.ah.gov.cn/

Fujian Province：https://www.fujian.gov.cn/

Jiangxi Province：https://www.jiangxi.gov.cn/

Shandong Province：http://www.shandong.gov.cn/

Henan Province：https://www.henan.gov.cn/

Hubei Province：https://www.hebei.gov.cn/

Hunan Province：https://www.hunan.gov.cn/

Guangdong Province：https://www.gd.gov.cn/

Hainan Province：https://www.hainan.gov.cn/

Sichuan Province：https://www.sc.gov.cn/

Guizhou Province：https://www.guizhou.gov.cn/

Yunnan Province：https://www.yn.gov.cn/

Shaanxi Province：https://www.shaanxi.gov.cn/

Gansu Province：https://www.gansu.gov.cn/

Qinghai Province：http://www.qinghai.gov.cn/

Inner Mongolia Autonomous Region：https://www.nmg.gov.cn/

Guangxi Zhuang Autonomous Region：http://www.gxzf.gov.cn/

Xizang Autonomous Region：https://www.xizang.gov.cn/

Ningxia Hui Autonomous Region：https://www.nx.gov.cn/

Xinjiang Uyghur Autonomous Region：https://www.xinjiang.gov.cn/

Beijing：https://www.beijing.gov.cn/

Tianjin：https://www.tj.gov.cn/

Shanghai：https://www.shanghai.gov.cn/

Chongqing：https://www.cq.gov.cn/
